# Neuronal effects of glabellar botulinum toxin injections using a valenced inhibition task in borderline personality disorder

**DOI:** 10.1038/s41598-022-17509-0

**Published:** 2022-08-20

**Authors:** Tillmann H. C. Kruger, Jara Schulze, Agnès Bechinie, Insa Neumann, Stefanie Jung, Christian Sperling, Jannis Engel, Antje Müller, Jonas Kneer, Kai G. Kahl, Matthias Karst, Julian Herrmann, Larissa Fournier-Kaiser, Liza Peters, Frank Jürgensen, Matthias Nagel, Welf Prager, Birger Dulz, Peter Wohlmuth, Volker Heßelmann, Christopher Sinke, M. Axel Wollmer

**Affiliations:** 1 Division of Clinical Psychology and Sexual Medicine, Department of Psychiatry, Social Psychiatry and Psychotherapy, Hannover Medical School, Carl-Neuberg-Str. 1, 30625 Hannover, Germany; 2 Center for Systems Neuroscience, Hanover, Germany; 3 Asklepios Clinic North-Ochsenzoll, Asklepios Campus Hamburg, Medical Faculty, Semmelweis University, Hamburg, Germany; 4 Asklepios Clinic North-Ochsenzoll, Clinic for Geriatric Psychiatry, Langenhorner Chaussee 560, 22419 Hamburg, Germany; 5 Hannover Medical School, Department of Psychiatry, Social Psychiatry and Psychotherapy, Hannover, Germany; 6 Hannover Medical School, Department of Anesthesiology and Intensive Care Medicine, Pain Clinic, Hannover, Germany; 7 Department of Psychiatry and Psychotherapy, Asklepios Clinic North-Wandsbek, Hamburg, Germany; 8 Prager & Partner, Dermatologische Praxis, Hamburg, Germany; 9 Asklepios Clinic North-Ochsenzoll, Clinic for Personality Disorders and Trauma, Hamburg, Germany; 10 Asklepios Proresearch, Hamburg, Germany; 11 Department of Neuroradiology, Asklepios Clinic North-Heidberg, Hamburg, Germany

**Keywords:** Neuroscience, Psychology

## Abstract

Previous studies have indicated that glabellar botulinum toxin (BTX) injections may lead to a sustained alleviation of depression. This may be accomplished by the disruption of a facial feedback loop, which potentially mitigates the experience of negative emotions. Accordingly, glabellar BTX injection can attenuate amygdala activity in response to emotional stimuli. A prototypic condition with an excess of negative emotionality and impulsivity accompanied by elevated amygdala reactivity to emotional stimuli is borderline personality disorder (BPD). In order to improve the understanding of how glabellar BTX may affect the processing of emotional stimuli and impulsivity, we conducted a functional magnetic resonance imaging (fMRI) study. Our hypotheses were (1) glabellar BTX leads to increased activation in prefrontal areas during inhibition performance and (2) BTX decreases amygdala activity during the processing of emotional stimuli in general. Using an emotional go-/no-go paradigm during fMRI, the interference of emotion processing and impulsivity in a sample of n = 45 women with BPD was assessed. Subjects were randomly assigned to BTX treatment or serial acupuncture (ACU) of the head. After 4 weeks, both treatments led to a reduction in the symptoms of BPD. However, BTX treatment was specifically associated with improved inhibition performance and increased activity in the motor cortex. In addition, the processing of negative emotional faces was accompanied by a reduction in right amygdala activity. This study provides the first evidence that glabellar BTX injections may modify central neurobiological and behavioural aspects of BPD. Since the control treatment produced similar clinical effects, these neurobiological findings may be specific to BTX and not a general correlate of symptomatic improvement.

## Introduction

There is accumulating evidence that botulinum toxin injections (BTX) into the glabellar region may alleviate the symptoms of major depressive disorders^1–5^. A meta-analysis has demonstrated that botox decreases depressive symptoms (d = 1.47) and is more efficient than placebo (d = 0.63)^6^, although they may be inflated by methodological issues such as blinding procedures. Earlier studies had already indicated a modulation of emotion processing and experiencing after aesthetically motivated glabellar BTX injections^7,8^.

The mechanism of action of glabellar or facial BTX injection on mood states has not yet been elucidated. A favoured explanation of the emotion modulating effects of facial BTX is the facial feedback hypothesis (FFH), which originates from early observations in the nineteenth century by Charles Darwin^9^. According to the FFH, the facial expression of an emotion causes a proprioceptive feedback to the brain, potentially facilitating a change from an initially faint, cool or semi-cognitive sensation to a powerful and sensually warm experience^10–12^. However, experimental evidence for the impact of facial expressions on subjectively perceived emotions, especially on the original ‘pen in the mouth’ condition is weak^13,14^ and the influence is smaller than initially thought^15^. Glabellar injections of BTX paralyse the corrugator supercilii and procerus muscles, which are key muscles in the expression of negative emotions, and thereby interrupt the feedback loop that would maintain and reinforce these emotions^7^. Patients with depression may have a relative overactivity of these “grief” muscles that might be corrected by BTX injections^16^. On a neuronal level, there is initial evidence of a reduction of amygdala reactivity in response to negative emotional stimuli after glabellar BTX injections in healthy subjects^17,18^.

A prototypic disease with an excess of negative emotions and impulsivity is borderline personality disorder (BPD^19^). BPD patients show enhanced activity of the corrugator muscles in response to the presentation of faces with negative emotional expressions^20^. The basic processing of emotions in BPD is usually biased towards the perception of negative emotions^21^ and patients show a decreased detection threshold for negative facial expressions^22^. Etiologically, the altered emotional processing and problems with emotion regulation may be associated with decreased behavioural inhibition^23,24^. The deficit in generating behavioural inhibition may manifest clinically as impulsivity, e.g. self-harming or suicidal behaviour and substance abuse^25^. Altered processing of emotional cues is consistently associated with an over-responsiveness of the amygdala in BPD^26–28^. Neuronal correlates of impulsivity are less consistent and may include the prefrontal and parietal areas^24^, with inhibition processes being primarily located in prefrontal areas^25^.

Preliminary data indicated that glabellar BTX injections may successfully treat the symptoms of BPD, including excessive negative emotionality and impulsivity^29^. Consequently, a randomised controlled trial (RCT) on the clinical effects of BTX in BPD was initiated (NCT02728778). As an experimental part of the RCT using a previously established emotional go/no-go paradigm^23^, we investigated the neurobiological mechanisms of glabellar BTX injections on the processing of emotional stimuli and impulsivity. Using functional magnetic resonance imaging (fMRI), we assessed two separate hypotheses: (1) glabellar BTX can lead to increased activation in prefrontal areas during inhibition performance and (2) the neurotoxin decreases amygdala activity during the processing of emotional stimuli in general, both in comparison to minimal acupuncture treatment.

## Results

### Clinical data

BPD symptoms significantly decreased over time by 6.11 points (BTX 5.67, ACU 6.62) in both groups as assessed by expert rating using the ZAN-BPD scale (F(1,42) = 44.71, p < 0.001, η^2^ = 0.51). There were no significant differences between the interventions (F(1,42) = 0.30, p = n.s.) and no interaction effect (F(1,42) = 0.25, p = n.s.). BSL-23 scores depicted a similar pattern over time with an average decrease of 0.58 points (BTX 0.58, ACU 0.58) (F(1,41) = 20.24, p < 0.001, η^2^ = 0.33), but no significant differences between the groups (F(1,41) = 0.86, p = n.s.) and no interaction effects (F(1,41) = 0.000, p = n.s.) (see Table 1). Finally, the H2A also showed a decrease over time of 7.58 points (BTX 9.68, ACU 5.38) (F(1,41) = 15.17, p < 0.001, η^2^ = 0.27), with neither group differences (F(1,41) = 0.06, p = n.s.) nor interaction effects (F(1,41) = 1.24, p = n.s.).

**Table 1:** Demographic and psychometric characteristics: (A) Demographic characteristics and the test statistics at baseline separate for BTX and ACU (total *N* = 45). (B) The scores of the ZAN-BPD, BSL-23 and H2A at baseline (T0) and at 4-week follow-up (T1) separate for BTX and ACU and their test statistics (total *N* = 45).

(A)					
Demographic variables	BTX group, n = 24	ACU group, n = 21	p-value		
	M ± SD or n (%)	M ± SD or n (%)			
**Age, in years**					
	28.75 ± 5.93	26.62 ± 4.61	0.19^a^		
**Highest level of education**					
Primary school	0 (0.0)	2 (9.5)			
Lower secondary school	2 (8.3)	2 (9.5)			
Intermediate secondary school	8 (33.3)	10 (47.6)			
Advanced secondary school	14 (58.3)	7 (33.3)	0.22^b^		
**Handedness, n (%)**					
Right	20 (83.3)	18 (85.7)			
Left	4 (16.7)	3 (14.3)	1.00^b^		
**Comorbidity, n (%)**					
Yes	24 (100.0)	20 (95.2)	0.47^b^		
Affective disorder	9 (37.5)	6 (28.6)	0.75^b^		
Anxiety disorder or obsessive compulsive disorder	12 (50.0)	14 (66.7)	0.37^b^		
Alcohol abuse	7 (29.2)	6 (28.6)	1.00^b^		
**Current psychotropic medication, n (%)**					
Yes	20 (83.3)	13 (61.9)	0.18^b^		

(B)					
Psychometric variables	BTX group, n = 24		ACU group, n = 21		p-value (group / time / group × time)
	T0 M ± SD	T1 M ± SD	T0 M ± SD	T1 M ± SD	
ZAN-BPD	16.04 ± 6.37	10.35 ± 4.75	15.76 ± 5.38	9.14 ± 5.10	0.59 / <0.001 / 0.62^c^
Affect	6.13 ± 2.32	4.13 ± 2.36	5.90 ± 2.43	3.95 ± 2.60	0.73 / <0.001 / 0.96^c^
Cognition	4.13 ± 2.07	2.74 ± 1.86	3.67 ± 2.46	2.48 ± 1.84	0.50 / <0.001 / 0.76^c^
Impulsivity	2.70 ± 1.69	2.00 ± 1.73	2.14 ± 1.11	1.00 ± 1.00	0.04 / <0.001 / 0.33^c^
Relationship	3.09 ± 2.19	1.48 ± 1.28	4.05 ± 1.50	1.71 ± 1.35	0.15 / <0.001 / 0.20^c^
BSL-23	2.16 ± 0.66	1.58 ± 0.91	2.36 ± 0.77	1.78 ± 0.89	0.35 / <0.001 / 0.99^c^
H2A	34.68 ± 14.31	25.00 ± 15.72	33.38 ± 12.88	28 ± 9.19	0.81 / <0.001 / 0.27^c^

*BTX* incobotulinumtoxinA, *ACU* acupuncture, *M* mean, *SD* standard deviation, *BTX* incobotulinumtoxinA, *ACU* acupuncture, *M* mean, *SD* standard deviation, *T0* baseline, *T1* 4-week follow-up, *ZAN-BPD* Zanarini Rating Scale for Borderline Personality Disorder, *BSL-23* Borderline Symptom List—23, *H2A* Hamburg-Hannover Agitation Scale. ^a^statistical analysis: *t*-test, ^b^statistical analysis: Fishers exact test, ^c^statistical analysis: repeated measures analysis of variance.

### Results of inhibitory performance hypothesis

#### Behavioural data

At a behavioural level, there were no differences between the groups regarding their average sensitivity [d′] for angry and happy pictures at baseline (angry: U = 239.000, Z = − 0.315, p = n.s.; happy: U = 235.500, Z = − 0.387, p = n.s., Mann–Whitney U test). For the BTX condition, sensitivity for angry pictures increased from T0 to T1 (Z = − 2.384, p = 0.017, η_p_^2^ = 12.16, Wilcoxon signed-rank test), whereas in the ACU condition this effect did not occur (Z = − 0.334, p = n.s.). For the happy picture condition, no time effects were seen in either groups.

**Figure 1:**
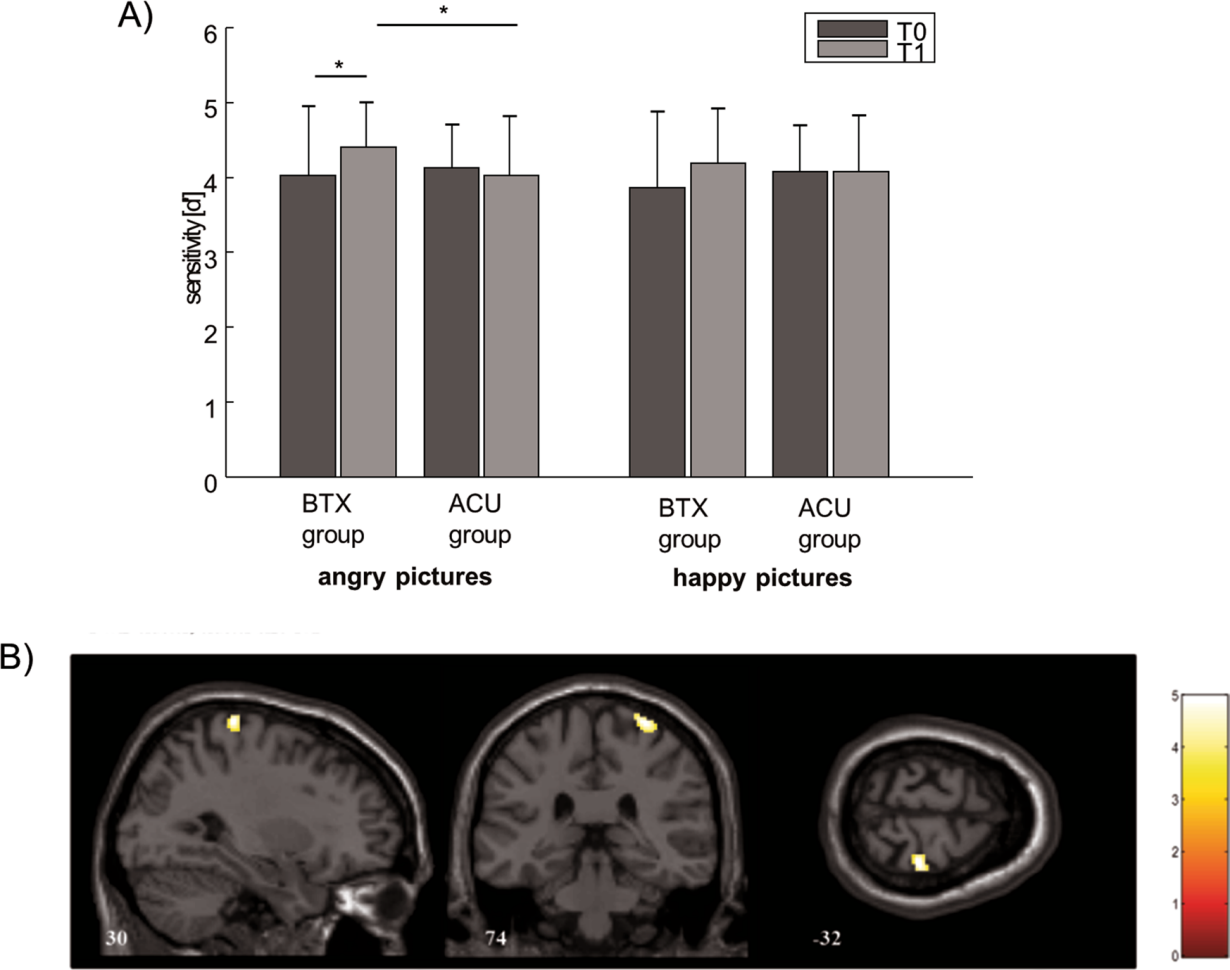
Behavioral effects of BTX and ACU in the emotional go-/no-go task and brain activation of the GO/NO-GO × TIME × GROUP interaction for angry pictures: (**A**) Shown are the pre- and post-scores of sensitivity [d′], separately for angry and happy pictures. (**B**) BOLD activations (FWE corrected on a cluster level) of the primary motor cortex (no-go > go) over time (T1 > T0) in the BTX group compared to the ACU group. For visualization results are overlaid on a T1-weighted image.

Further analyses of group differences at T1 using Mann–Whitney U tests revealed higher sensitivity in the BTX group compared to the ACU group, again for the angry picture (U = 164.500, Z = − 2.250, p = 0.024, η^2^ = 0.088) but not for the happy picture condition (U = 231.500, Z = − 0.503, p = n.s.; see Fig. 1A).

#### ROI and whole brain data

To assess the impact of BTX on behavioural inhibition, the no-go > go contrasts as an indicator of change in inhibitory performance over time (T1[no-go > go] > T0[no-go > go]) was evaluated for the inferior frontal gyrus and the pre-SMA as ROIs and additionally on the whole brain level. For the ROI analysis, the angry picture contrast revealed higher activation of the inferior frontal gyrus over time in the BTX group in comparison to the ACU group. No results were found for the pre-SMA. On the whole brain level over time, the precentral gyrus (primary motor cortex) of the BTX group showed higher activation in comparison to the ACU group for the angry picture condition (see Fig. 1B and Table 2). Both analyses were also performed for the happy picture condition, which did not yield any significant results.

**Table 2:** BOLD activation peaks: shown are the peak activations, cluster size, t-value and corresponding AAL labels for the following contrasts. (A) Peaks in the direct contrast BTX > ACU. (B) Peaks for the BTX group only.

(A)			
Region	BTX > ACU		
	T	CS	Peak coord
**Inhibitory-related neuronal activation (angry [T1 nogo > go] > [T0 nogo > go])**			
Inferior frontal gyrus, left	5.01*	65	− 40 30 − 10
Precentral gyrus, right	4.96	172	30 − 32 74

(B)			
Region	BTX		
	T	CS	Peak coord
**Processing-related neuronal activation (happy T0 > T1)**			
Amygdala, right	5.08*	36	26 − 4 − 20
Amygdala, left	4.69*	46	− 26 − 8 − 18
**Processing-related neuronal activation (angry T0 > T1)**			
Amygdala, right	3.81*	8	28 − 8 − 20

*BTX* incobotulinumtoxinA, *ACU* acupuncture, *CS* cluster size, Peak coordinates of clusters are according to the MNI-space (Montreal Neurological Institute), *T* t-score of peak voxel, FWE correction with p < 0.05 at whole-brain level, T-values marked with * are corrected using small volume correction (SVC) in a-priori regions of interest (see “Methods”) at an FWE corrected level of p < 0.05.

### Results of emotional processing hypothesis

#### ROI and whole brain data

**Figure 2:**
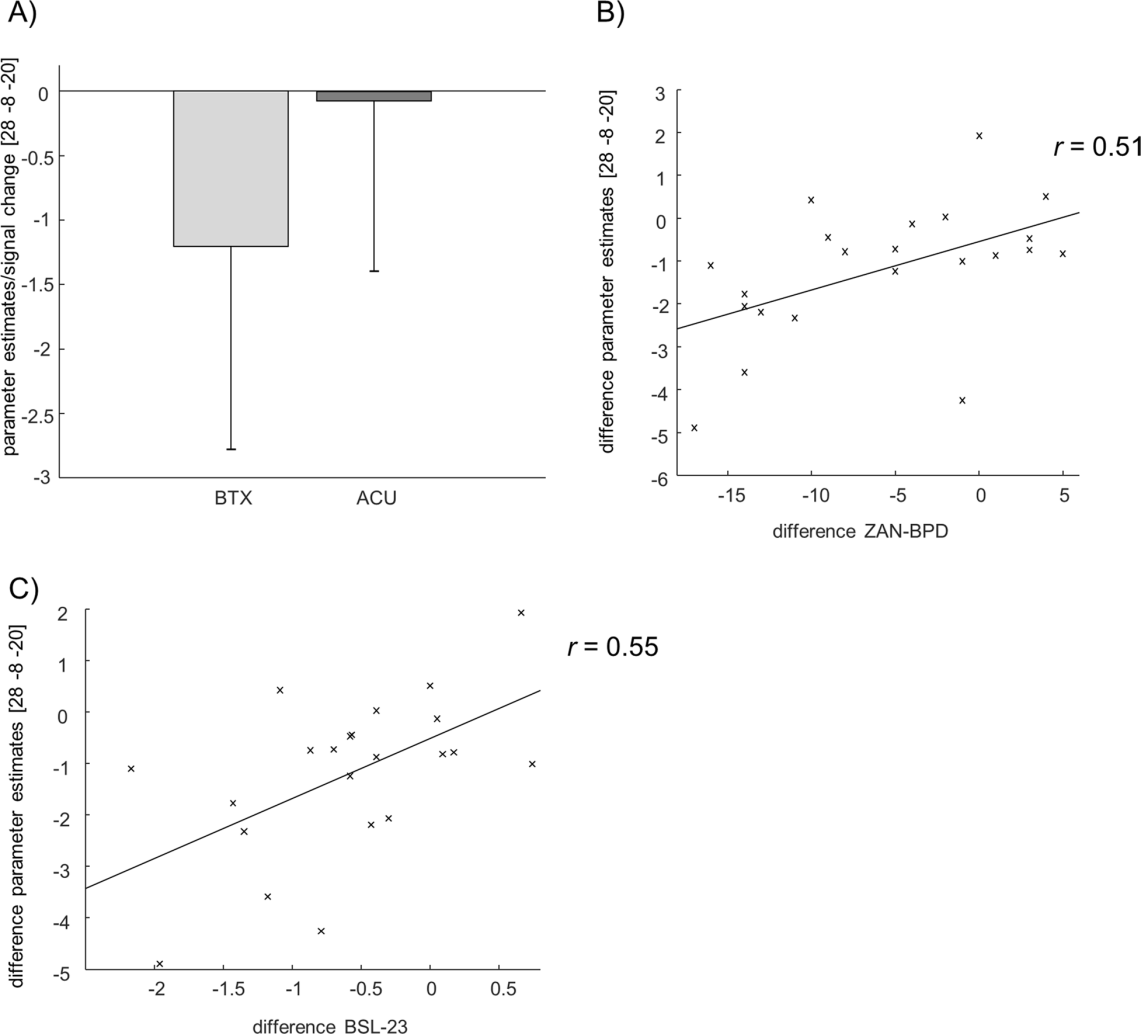
Brain activation of the amygdala at angry pictures: (**A**) Change of parameter estimates in the right amygdala for the BTX and ACU group. (**B**) Correlation between the difference of the parameter estimates in the amygdala and the difference of ZAN-BPD for BTX. (**C**) Correlation between the difference of the parameter estimates in the amygdala and the difference of BSL-23 for BTX.

For the conducted ROI analysis of both amygdalae during the presentation of affective stimuli, a direct comparison between groups and times (BTX [T1 > T0] > ACU [T1 > T0]) did not yield any significant results, neither for angry nor for happy pictures. Regardless, examining the angry pictures over time for each group separately indicated a decrease in the activation of the right amygdala within the BTX but not the ACU group (see Fig. 2A). Moreover, extracted parameter estimates of the cluster in the right amygdala showed a positive correlation with the change in symptomatology as assessed by ZAN-BPD (r = 0.51, p = 0.015) and BSL-23 (r = 0.55, p = 0.011) in the BTX group only (for ZAN-BPD see Fig. 2B, for BSL-23 see Fig. 2C). Thus, a decrease in right amygdala activation was associated with a decrease in symptomatology after BTX treatment. A correlation analysis of d’ change of angry pictures did not show any significant results with the fMRI data (*r* = − 0.41 (p = n.s.)). For the happy picture condition, ROI analysis of the bilateral amygdalae showed a decrease of left and right amygdala activity over time, again only within the BTX group (for all results, see Table 2). On the whole brain level, no interaction effects could be detected.

## Discussion

This study investigated the neuronal effects of a single glabellar BTX injection in patients with BPD compared to an acupuncture control condition. After 4 weeks, both groups showed similar clinical improvement as assessed by standard expert (ZAN-BPD) and patient ratings (BSL-23). This finding is in line with a previous trial that also reported a relatively high placebo response compared to a psychotropic drug in BPD^30^. In contrast, behavioural and neuronal assessments of emotion processing and inhibition performance of this study revealed an indication for differences between the two treatments.

For our first hypothesis on the effect of BTX on inhibitory performance in BPD, we examined the behavioural data of the go/no-go paradigm and the fMRI data of the group (BTX vs. ACU) × time (T1 vs. T0) × condition (go vs. no-go) comparison on specific ROIs and on the whole brain level.

On the behavioural level, the increase in sensitivity and thus the decrease in wrong answers during no-go trials in the angry condition after BTX may be interpreted as an improvement of inhibition performance, which in turn may be due to lowered impact of negative emotions on inhibition processes. According to previous research, patients with BPD showed reduced sensitivity towards angry and happy faces compared to controls^23^ and the effects were even more pronounced for the happy picture condition. Effects of increased alertness during presentation of affective facial expressions may be a reason for this difference. In the current study, a certain normalisation or improvement of inhibitory performance after BTX treatment occurred. Based on the FFH, we argue that diminished facial expressions and/or reduced perception of the muscle tone eventually lead to blunted emotions elicited by the perception of emotional images, which in turn lead to a reduction of interference with inhibitory performance.

For the ROI analysis of the inferior frontal gyrus and the pre-SMA, the analysis revealed increased activity of the inferior frontal gyrus over time in the BTX group in comparison with the ACU group. These results are in line with previous literature, which suggests the involvement of the left and right inferior frontal gyrus in response inhibition^31,32^.

With respect to the interaction effects of emotion processing and inhibition performance in BPD^23,24^, the whole brain analysis detected an increased activity of parts of the right precentral gyrus during the angry picture condition after BTX treatment. The activation occurred in the area of representation of the upper extremities on the primary motor cortex (M1). Activation in this area was unexpected, as we had hypothesised prefrontal activation. Contrary to the usual assumption that the prevention of planned movement engages parts of the prefrontal cortex and the basal ganglia, some research suggests that M1 also shows an intracortex inhibitory network that seems to be important particularly in the final stage of response inhibition^33^: Specifically, movement decisions, made in pre-motor areas, can still be prevented in M1. The go/no-go paradigm used in this study was designed to trigger a sustained specific movement pattern, where the movement intention in some trials should be suppressed on short notice. A lack of intracortex inhibition of M1 and its connection to impaired movement prevention has been described in psychiatric disorders with difficulties in behavioural inhibition such as Tourette’s syndrome and attention deficit hyperactivity disorder^34,35^. Therefore, it can be assumed that the higher activation of M1 after BTX in the angry picture condition could be a sign of enhanced inhibitory control on movement patterns immediately before a respective action potential is generated by motor neurons and is sent to the spinal cord.

For our second hypothesis that BTX might have an impact on emotion processing in BPD we conducted a ROI analysis of the group (BTX vs. ACU) × time (T1 vs. T0) contrasts, regardless of the condition (go vs. no-go). The ROI analysis showed a reduction of right amygdala activation during the processing of angry faces, that occurred within the BTX group but not the ACU group, although a direct effect of the between group and time comparison was not significant. A reduction of right and left amygdala activity was also seen for the happy picture condition, when comparing the two time points within the BTX group, but not when contrasting time within the ACU group.

A wealth of studies have highlighted the role of the amygdala in processing highly salient emotions such as anger and fear, and a functional connection between amygdala and striatum has been described as important for generating coping behaviour in response to negative emotions (see Ref.^36^ for review). For BPD and other psychiatric disorders, this interrelationship is assumed to be intensified compared to healthy controls and is seen as a general marker for psychopathology^37^. Interestingly, some studies showed an aberrant connectivity^37–39^ and higher activation of right amygdala during processing of fearful stimuli^40^ in BPD, thus one could speculate that the reduction in the right amygdala after treatment with BTX as observed here may represent a kind of normalization.

The deactivation of the right and left amygdala during the happy face condition after BTX treatment seems counterintuitive at first glance because we did not address muscles of positive emotional expression such as the orbicularis oculi, risorius or major zygomatic muscles. However, it is possible that small amounts of the incobotulinumtoxinA paralyzed parts of the orbicularis oculi by diffusion and thereby affected the processing of positive emotions. Moreover, facial EMG studies in BPD patients did not show increased activity of corrugator supercilli and procerus muscles in response to positive facial expressions^20^. However, there are findings of general negative affectivity in patients with BPD^21^, a bias toward interpreting neutral faces as negative^21^, a general intensified activation of the amygdala in response to happy faces^26,41^, and a general reduction of sensitivity measures in an emotional go/no-go paradigm irrespective of valence^23^. Therefore, the current findings may indicate that a reduced expression and/or perception of negative emotions may lead to a normalisation of altered emotion processing, which includes those of positive valence.

### Strengths and weaknesses

The study was only conducted in female participants. A generalisation to male patients with BPD is therefore limited and poses an opportunity for future research. Furthermore, the usage of psychotropic medication could not be controlled in the sample, since the participants varied greatly in terms of the prescribed compound and dose. It should be additionally noted that although comorbid disorders dominating the clinical appearance were an exclusion criteria, usually a high number of patients with BPD show symptoms of axis-I disorders^42^. Because of the variety and differing severity of secondary comorbid disorders in this study, we could not control for them, thus the results are limited. As we did not exclude participants with attention deficit hyperactivity disorder (ADHD) nor assessed ADHD with psychometric tools, we could not control for its influence on our effects. During the screening, two women in the ACU and one woman in the BTX indicated a lifetime diagnosis of ADHD and one of them disclosed the use of typical ADHD medication.

For the second hypothesis, we were only able to show a time effect for the BTX group. Thus, it is not clear if the effects show a therapeutic effect of BTX in BPD or if it is a general BTX finding. For further research, a non-patient control group would be meaningful.

## Conclusion

This is the first study to investigate a novel approach to the peripheral manipulation of emotional expression in an undertreated disorder (BPD), combining clinical data with neuroimaging. The findings contribute to the highlighted role of the amygdala in emotional processing and its possible connection to impulsivity in BPD and support the idea of embodied emotions and their modulation by glabellar BTX injections. Despite the absence of a clear clinical benefit of BTX on BPD symptomatology compared to ACU in this study, these results may encourage further research into the use of BTX in the treatment of BPD and other mental disorders involving an excess of negative emotionality.

## Methods and materials

This functional MRI study is part of a larger randomised clinical trial (RCT) which was conducted from August 2016 through February 2019 at Hannover Medical School and Asklepios Clinic North—Ochsenzoll in Germany (ClinicalTrials.gov Identifier: NCT02728778). Primary and secondary clinical outcomes are described elsewhere (Ref.^43^). The subjects were randomly allocated to a single BTX treatment of the glabellar region or a control intervention, which consisted of acupuncture treatment (ACU) of the head for about 20 min at week 0 and 2. The control condition diverged from previously used saline injections because prior studies have hypothesised the smaller placebo effects in comparison to BTX might be due to unfulfilled expectations due to unblinding. We implemented a minimal acupuncture treatment of the head as a control condition with respect to good comparability in terms of (a) treatment type (needles) and (b) treatment site (head). Although considered a treatment option for psychiatric disorders, as some authors have reported significant psychotropic effects, the support for acupuncture for psychiatric indications remains insufficient due to the low quality of the data high risk of bias^44-48^. it was therefore chosen as a control condition in this study.

The MRI data were collected at baseline (T0) and four weeks after baseline (T1) to evaluate the immediate paralytic effects of BTX injections rather than the mid-term impact. Additionally, clinical data was also assessed at one visit between T0 and T1. The study was conducted in accordance with the Declaration of Helsinki and was approved by the leading ethics committee at the Hannover Medical School. After patients were informed about the random allocation to either the BTX or ACU group, they gave written informed consent to participate. They were free to withdraw from the study at any time and received monetary compensation for each MRI visit. To control for the effects of disappointment, all subjects were offered to receive the other treatment after completing the clinical trial.

### Participants

Participants were recruited via public advertisements, social media and in outpatient units of the two study sites. In total, 45 women were deemed eligible according to the inclusion and exclusion criteria and gave consent for the MRI assessment (n = 24 in BTX group and n = 21 in ACU group). The groups did not show any significant differences regarding age, education years, handedness, number of comorbidities or current medication (Table 1). A detailed description of the sample and clinical effects can be found in Wollmer et al.^43^.

Inclusion criteria were a current diagnosis of BPD according to the International Classification of Diseases (10th ed.; ICD10^49^) and the Structured Clinical Interview for Axis II DSM-IV disorders (SCID-II^50^), conducted by trained study personnel; age between 18 and 40 years and a stable treatment for at least 8 weeks prior to and after enrolment (i.e. no qualitative or quantitative changes in medication or psychotherapy). Women with a manifest comorbid psychiatric disorder dominating the clinical impression except psychoactive substance abuse (ICD-10 F1x.1) and mild depressive episodes (ICD-10 F32.0 or F33.0) were excluded. Especially in case of major depression this strict exclusion was necessary in order to restrain confounding effects of the treatment on the comorbid disorders, as it was previously shown that BTX injection in the glabellar region has an beneficial effect on depressive symptoms^6,51^.

### Clinical interventions, randomisation and blinding

The BTX group received an injection of 34 U incobotulinumtoxinA (Bocouture^®^, Merz, Germany), dissolved in 0.9% NaCl solution (100 U/2.5 ml) using a 30G needle by a trained physician after baseline assessments at T0. The injection sites consisted of five intramuscular points in the glabellar region: 8U were applied to the procerus muscle, 7U bilaterally to the medial parts and 6 U the lateral parts of the corrugator supercilii muscle. For the ACU group, acupuncture needles (B-type needle, acupuncture needle, No. 3, 0.20 × 15 mm, Seirin) were placed by either a trained physician or a study nurse with special expertise in acupuncture after baseline assessment (T0) and 2 weeks after. The acupuncture scheme was designed by an acupuncture expert and comprised Yintang, BI2 paired, GB14 and Du20 at the forehead and apex of the head. Essentially, because of only two dates of administration with a long interval in between (2 weeks), short needle retention time (20 min), an intensity at the lower limit for any conceivable specific acupuncture effect can be assumed and thus ACU treatment may be regarded as a “minimal acupuncture” intervention. Acupuncture was chosen as control condition in order to avoid disappointment of the participants, thereby decrease dropout rates, and prevent nocebo effects that may arise from discovering allocation to the placebo group.

Regardless of the allocated group, the participants spent around 30 min with the clinician at each visit before the assessment of the clinical data to avoid deblinding the rater. This was necessary as the different treatments had different durations (BTX injection around 5 min acupuncture around 30 min) and otherwise the rater could guess the treatment based on the treatment time. The randomisation procedure was conducted by a third party (ASKLEPIOS proresearch) that was not involved in the practical execution of the study, using variable block length. Blinding of the participants was difficult due to the obvious and perceivable paralytic effects of BTX injections. The study design was single blinded; participants and the treatment administrators were aware of the allocated intervention, while the psychometric raters remained blind throughout the study. To prevent their unblinding, participants wore a cap covering their forehead during all visits and were instructed to keep confidentiality about their treatment.

### Psychometric assessments

BPD symptomology was assessed on the Zanarini Rating Scale for Borderline Personality Disorder (ZAN-BPD^52^). For the self-evaluation of BPD symptoms, the Borderline Symptom List 23 (BSL-23^53^) was used. In addition, agitation as an important clinical feature of BPD was measured using the Hamburg-Hannover Agitation Scale (H2A^54^).

### Experimental procedure, paradigm and stimuli

We were specifically interested in the influence of glabellar BTX injections on emotion processing in general and the interaction between inhibitory performance and emotional processing. Therefore, we used an established valenced go/no-go paradigm^23^. Participants were asked to respond as fast as possible to geometric figures (squares or circles) overlaid on faces displaying a happy or angry emotion^55^. Presentation^®^ was used for the stimuli display and the recording of behavioural data (Presentation 16.3, Neurobehavioral Systems Inc, Berkley, CA, USA; www.neurobs.com). Stimulus material was shown on a 32″ monitor from NordicNeuroLab (NNL; Bergen, Norway; www.nordicneurolab.com) placed at the foot of the subjects which was visible via a mirror. Behavioural data was collected using response grips from NNL in the right hand of the participants. The pictures span a visual angle of 6.6° × 9°, while the geometrical figures had a visual angle of 0.5° overlaid on the nose region of the faces.

The visual stimuli were extracted from the NimStim Set of Facial Expressions^55^. The facial expressions implied open emotions (mouth open) to facilitate recognition and decrease the scope for interpretation. Per condition (angry/happy), pictures of 15 female and 15 male faces were shown. Each condition was displayed three times, resulting in a total of 90 angry faces and 90 happy faces. The faces were displayed for 2 s, followed by a variable break between four and eight seconds (6 s on average). Participants were instructed to press a button as fast as possible whenever a black circle appeared (65 trials/condition, 72% of pictures) and suppress a reaction whenever a black square appeared instead (25 trials/condition, 28% of pictures). The experiment started with a training phase where only go cues were presented in 30 trials using faces with closed expressions (i.e. mouth closed). Overall, the experiment took 24 min to complete.

### fMRI data acquisition and processing

For MRI data acquisition, a Siemens 3 T Skyra running Syngo VE11 with a standard 64 channel head coil was utilised. Using a gradient simultaneous multislice EPI T2* sensitive sequences, a total of 84 axial slices (resolution 2 × 2 × 2 mm) per volume were acquired in interleaved ascending order. The following parameters were applied: repetition time (TR) = 1.55 s, echo time (TE) = 32 ms, flip angle = 90°, field of view = 256 × 256 mm, acceleration factor = 4. Using a T1 weighted magnetisation prepared rapid acquisition gradient echo sequence, an individual high resolution anatomical image was acquired for each participant prior to functional scans (resolution 0.9 × 0.9 × 0.9 mm, TR = 2.3 s, TE = 3 ms, flip angle = 9°, 255 × 270 mm). Using dcm2nii (McCausland Center for Brain Imaging, University of South Carolina), DICOM images were converted to NIFTI format. Functional scans were realigned after removing the first five scans to compensate for T1 saturation effects. Afterwards, the mean echo planar image was co-registered to the individual T1 images. Using SPM12 (Functional Imaging Laboratory, Wellcome Trust Centre for Neuroimaging Institute of Neurology, UCL), structural and functional images were normalised to MNI space with a voxel size of 2 × 2 × 2 mm and smoothed with a 4 × 4 × 4 mm FWHM Gaussian kernel.

### Statistical analyses

#### Analysis of clinical data

Clinical data (ZAN-BPD, BSL-23 and H2A) were analysed using SPSS© (Version 26, IBM Inc.). Two-tailed testing and a p-value ≤ 0.05 was chosen for statistical significance. According to Kolmogorov–Smirnov test the data was normally distributed and thus parametric testing was chosen. A 2 × 2 repeated measures analysis (rmANOVA) was conducted to test for intervention and time differences as well as time by intervention interactions on the outcomes of ZAN-BPD, BSL-23 and H2A. INTERVENTION (BTX/ACU) was entered as the between-subject factor and TIME (T0/T1) as the within subject factor. To determine correlations between functional and clinical data, the Pearson correlation coefficient was chosen and Bonferroni corrected because of multiple comparisons. Consequently, a p-value ≤ 0.017 was considered as statistically significant.

#### Analysis of behavioural data

Behavioural data was analysed with SPSS© (Version 26, IBM Inc.). Statistical analyses were performed using two-tailed testing. For dependent variables, the correct go responses and the incorrect no-go responses were calculated. To account for possible individual answer strategies and decrease response bias, we chose to report sensitivity (d′) instead of commission errors; d′ is calculated by subtracting the z-transformed false alarm rate (incorrect no go responses) from the z-transformed hit rate (correct go responses). Values of 0 were transformed into 0.01 and values of 1 into 0.99. As the Kolmogorov–Smirnov test revealed deviations from the normal distribution, non-parametric testing was chosen. Wilcoxon signed-rank tests were performed and Bonferroni corrected because of multiple testing. Thus a p-value ≤ 0.025 was considered as statistically significant.

#### Analysis of fMRI data

Data analysis was performed using the general linear model (GLM). For the final analysis, three subjects (2 BTX, 1 ACU) had to be excluded due to head movement > 3 mm, resulting in a sample of n = 42 for the final analysis. The variables age and H2A were entered into the GLM as covariates. Age is a parameter often included in models, due to its known effect on fMRI analysis^56^. Since research suggests that agitation potentially modulates the relationship between BTX treatment and clinical outcomes^57^ and agitation is a known symptom of BPD, we included the H2A total score as a covariate.

On a subject level, the two time points (T0 and T1) were modelled using four regressors of interest (angry/go, angry/no-go, happy/go and happy/no-go) as well as six regressors of no interest (movement parameters in the scanner) for each time point. Each boxcar stimulus function was convolved with a canonical hemodynamic response function. The data was then high-pass filtered with a cut-off period of 128 s. To set up a random effects analysis, the contrast images of main and interaction effects were used at the group level. Afterwards, a two-sided t-test was computed to identify group differences. On the cluster level, a threshold for all analyses was set to p ≤ 0.05 family wise error (FWE), corrected for multiple comparisons. Further, an extent threshold of five voxels was applied. The peak voxel of significant clusters was localised using automatic anatomical labelling (AAL^58^).

For our first hypothesis, which tested the effects of BTX on inhibitory performance, a region-of-interest (ROI) analysis and a whole-brain analysis for the happy and angry picture contrasts of time (T1 vs. T0) × condition (no vs. no-go) (e.g. (angry no-go T1 > angry go T1) > (angry no-go T0 > angry go T0)) was performed. As the inferior frontal gyrus and the pre-supplementary motor area (pre-SMA) are thought to be involved in the inhibition of responses^32^, they were defined as ROIs, applying a small volume correction^59,60^.

Regarding our second hypothesis on the effect of BTX on emotional processing, we conducted a ROI analysis on the group (BTX vs. ACU) × time (T1 vs. T0) contrasts for the emotional pictures regardless of the condition (e.g. angry go + angry no-go for T1 > angry go + angry no-go for T0). Based on the a priori hypothesis, the amygdala was defined as an ROI. This structure was chosen due to evidence of increased activation in patients with BPD^24^ and the reported inhibitory effect of glabellar botulinum toxin injections on amygdala function^17,18^. The anatomical definition was used from the AAL atlas^58^. Parameter estimates were extracted using the eigenvariate function in SPM.

## Data Availability

The datasets generated during and/or analysed during the current study are available from the corresponding author on reasonable request.
